# Cyclic stretch increases glucose uptake and pro-inflammatory response in rat alveolar epithelial type II cells

**DOI:** 10.3389/fphys.2026.1909536

**Published:** 2026-10-01

**Authors:** Jorge M. C. Ferreira, Robert Huhle, Sabine Müller, Thea Koch, Marcelo Gama de Abreu

**Affiliations:** 1 Department of Anesthesiology and Intensive Care Medicine, Pulmonary Engineering Group, University Hospital Carl Gustav Carus Dresden at Technische Universität Dresden, Dresden, Germany; 2 Division of Intensive Care and Resuscitation, Department of Anesthesiology, Integrated Hospital Care Institute, Cleveland Clinic, Cleveland, OH, United States; 3 Outcomes Research Consortium, Department of Anesthesiology, Integrated Hospital Care Institute, Cleveland Clinic, Cleveland, OH, United States; 4 Department of Cardiothoracic and Vascular Anesthesia and Intensive Care, Medical University Graz, Graz, Austria

**Keywords:** glucose uptake, inflammation, type II alveolar epithelial cells, ventilator-induced lung injury, VILI

## Abstract

**Background:**

Mechanical ventilation (MV) is used to improve gas exchange but can exacerbate lung inflammation by increasing alveolar cell stretch, leading to ventilator-induced lung injury (VILI). Alveolar epithelial stretching promotes cytokine release, but its effects on glucose uptake remain unclear. *In vitro* studies suggest that high tidal volume ventilation corresponds to cyclic stretch levels of approximately 18%, whereas larger deformations represent supraphysiological and injurious conditions. Therefore, 30% cyclic stretch was used to reproduce severe alveolar deformation associated with volutrauma. We investigated the modulation of inflammation and glucose uptake in alveolar epithelial type II cells exposed to excessive cyclic stretch. We hypothesized that cells exposed to an overstretch of 30% at 1 and 4 hours would increase glucose uptake and pro-inflammatory responses.

**Methods:**

Rat alveolar epithelial type II cells (L2 AECs) treated and untreated with lipopolysaccharide (LPS) were exposed to 1 or 4 hours of 30% cyclic stretch. Non-stretched cells, stimulated or not with LPS, served as a control. Glucose oxidase activity was measured using a colorimetric/fluorometric assay, while glucose uptake was assessed by immunofluorescence using the fluorescent glucose analog 2-[N-(7-nitrobenz-2-oxa-1,3-diazol-4-yl)amino]-2-deoxyglucose (2-NBDG). Gene expression and secretion were measured by real-time polymerase chain reaction (RT-qPCR) and enzyme-linked immunosorbent assay (ELISA), respectively. Statistical analyses were performed using one-way ANOVA followed by Šidák’s multiple comparisons test, with significance set at *p* < 0.05.

**Results:**

30% cyclic stretch significantly increased glucose uptake and glucose oxidase activity in both LPS-treated and untreated cells. Inflammatory gene expression and mediator secretion were predominantly increased in LPS-primed cells, although the effects varied according to the marker and time point.

**Conclusions:**

Overall, excessive cyclic stretch increased the glucose-related readouts assessed in AECs L2 cells, whereas inflammatory responses were more strongly influenced by LPS stimulation and varied among mediators and time points.

## 1 Introduction

Mechanical ventilation (MV) is a life-saving therapy that aims to maintain adequate gas exchange and to reduce the work of breathing in intensive care patients. However, MV can damage the lungs or initiate lung injury, known as ventilator-induced lung injury (VILI) (Cruz et al., 2018; Botta et al., 2021; Ferreira et al., 2022; Ran et al., 2023). *In vivo* and *in vitro* studies have shown that excessive stretching of alveolar epithelial cells (AECs) can promote the expression and release of cytokines and other pro-inflammatory mediators, contributing to biotrauma (Rentzsch et al., 2017; Wang et al., 2017; Huhle et al., 2018; Ferreira et al., 2022; Ran et al., 2023). These processes contribute to the cascade of cellular and biochemical events involved in the pathogenesis of acute respiratory distress syndrome (ARDS) (Sipahi, 2014).

Alveolar epithelial type II cells, located on the alveolar wall, are essential for maintaining alveolar integrity and contribute to inflammatory responses within the alveolar epithelium (Crosby et al., 2011; Suki and Hubmayr, 2014; Tanaka et al., 2017). Mechanical stretch can increase the expression of pro-inflammatory mediators in these cells *in vitro* (Ferreira et al., 2022). However, considerably less is known about how excessive mechanical stretch affects glucose-related cellular responses and whether these responses are associated with inflammatory activation. Increased cellular glucose uptake may reflect the energetic requirements of cytoskeletal remodeling, maintenance of epithelial barrier function, transcriptional activation, protein synthesis, and inflammatory mediator production during mechanical stress. In addition, pulmonary uptake of the glucose analogue, 18F-FDG has been used to assess metabolically active inflammatory lung tissue. However, 18F-FDG uptake reflects the combined metabolic activity of multiple cell populations, including neutrophils, macrophages, endothelial cells, and epithelial cells. Therefore, it remains unclear whether increased glucose uptake by mechanically stretched alveolar epithelial cells directly reflects inflammatory activation or represents an independent metabolic response. A cyclic stretch magnitude of 30% was selected as an experimental model of severe alveolar overdistension. Physiological breathing produces relatively small changes in alveolar epithelial surface area, whereas high tidal volumes during mechanical ventilation are associated with excessive alveolar deformation, volutrauma, inflammatory signaling, and VILI. *In vitro* studies further suggest that high tidal volume ventilation corresponds to cyclic stretch levels of approximately 18%, whereas larger deformations above this range represent supraphysiological and injurious conditions. Thus, 30% cyclic stretch was used to model severe mechanical deformation above the range associated with injurious ventilation (Bigatello and Pesenti, 2009; Beitler et al., 2016; Samary et al., 2016; Pelosi et al., 2018). (Dreyfuss and Saumon, 1998; Beitler et al., 2016; Bates and Smith, 2018) and (Terragni et al., 2007; Neto et al., 2016) While our previous study investigated the contribution of static pre-stretch combined with dynamic stretch to inflammatory responses in L2 alveolar epithelial cells, the present study addresses a distinct question by assessing two glucose-related readouts, 2-[N-(7-nitrobenz-2-oxa-1,3-diazol-4-yl)amino]-2-deoxyglucose (2-NBDG) uptake and glucose oxidase activity, in parallel with inflammatory gene expression and mediator secretion under 30% cyclic stretch. These glucose-related measurements represent distinct experimental readouts and were not considered interchangeable. We hypothesized that exposure to 30% cyclic stretch for 1 and 4 hours would increase 2-NBDG uptake and alter inflammatory responses in alveolar epithelial type II cells, and we examined the patterns of these glucose-related and inflammatory responses across experimental conditions and time points.

## 2 Materials and methods

### 2.1 Cell culture preparation

L2 AECs (ATCC L2 cell line, CCL-149™) from rats, were grown until confluent on BioFlex six-well Collagen I coated plates (Flexcell International Corporation, Burlington, NC, United States) at a density of 1,3 x 10^5^ cells/well, in Dulbecco’s modified Eagle medium (DMEM, with High Glucose, L-glutamine, Phenol Red and Sodium Pyruvate, Gibco, # 11995073) supplemented with 10% fetal bovine serum (FBS, Gibco) and 50 μg/mL gentamycin sulfate (Gibco, # 15710049). Cells were incubated at 37 °C, at 6.5% CO_2_ for 24 hours. Cells were washed twice with sterile phosphate buffered saline (PBS) and incubated with DMEM (without Phenol Red, Merck Sigma-Aldrich, #D1145) containing 1% FBS, 50 µg gentamycin sulfate/mL, 4 mM L-alanyl-L-glutamine (Merck Sigma-Aldrich, # G8541), and 1mM Sodium Pyruvate (Gibco, # 11360070. Moreover, before the stretch experiments, cells were preincubated for 1 h with 2 µg/ml LPS (*Escherichia coli* O111:B4, SIGMA-Aldrich, St. Louis, USA).

### 2.2 Customized stretching device

The customized stretching device was used as described in previous studies (Rentzsch et al., 2017; Ferreira et al., 2022; Ran et al., 2023). Briefly, three cylindrical indenters applied homogeneous stretch to three membranes of a six-well BioFlex Culture Plate (Flexcell International Corporation, Hillsborough, USA), while the remaining three wells served as non-stretched controls. Vertical movement of the indenters was generated by a stepper motor (Maxon Motor AG, Sachseln, Switzerland). A custom LabVIEW program (National Instruments, Austin, TX, USA) was used to control the stepper motor and adjust the stretch duration, frequency, and amplitude (Rentzsch et al., 2017; Ferreira et al., 2022). The stretch group was exposed to 30% strain at a frequency of 0.5 Hz with a stretch/relaxation ratio of 1:1 (sinusoidal pattern) for 1 and 4 hours, **Supplementary Figure 1**.

### 2.3 Cell viability assay

Cell viability was assessed by measuring lactate dehydrogenase (LDH) activity in the culture supernatant using the Cytotoxicity Detection Kit Plus (Roche, Mannheim, Germany), according to the manufacturer’s instructions and as previously described (Dreyfuss and Saumon, 1998; Ferreira et al., 2022).

### 2.4 Glucose oxidase activity colorimetric/fluorometric assay kit

Glucose oxidase activity was measured using a BioVision assay kit (K788–100 CA, USA). Cells were homogenized by 100 - 200 μl of the glucose oxidase (GOx) Assay Buffer and centrifuged at 13,000g for 10 min to remove the insoluble material. Through 96 well plate, 1μl sample per well was added, and adjusted with a final volume of 50 μl of GOx Assay Buffer. A positive control was prepared in parallel, with 2μl of the GOx positive control sample (1 vial) and 50 μl of the GOx Assay Buffer. The reaction mix (GOx Assay Buffer 36 μl, GOx Developer 2 μl, OxiRed™ Probe 2 μl, GOx Substrate 10 μl) was prepared and added to each well. Plate was incubated for 5 min at 37 °C and measured the fluorescence (OD 570 nm) with a fluorescence reader (BioTeK, Bad Friedrichshall, Germany) at different period times (5 min, 17 min, 47 min, 1 h, and 2 hours).

### 2.5 RNA extraction

Total RNA was extracted with a peqGOLD Total RNA Kit (VWR, Dresden, Germany) and reverse transcribed with the qScript cDNA SuperMix (Beverly, USA). In both cases, according to the manufacturer’s instructions (Ferreira et al., 2022; Ran et al., 2023). RNA concentrations were evaluated using a spectrophotometer, and the purity was assessed based on the ratio of absorbance at 260 nm and 280 nm (A260–A280).

### 2.6 Gene expression

Gene expression of amphiregulin (AREG), interleukin-6 (IL-6), monocyte chemoattractant protein-1 (MCP-1), and macrophage inflammatory protein-2 (MIP-2) was analyzed by quantitative real-time polymerase chain reaction (qRT-PCR), as previously described in previous publications (Dreyfuss and Saumon, 1998; Bates and Smith, 2018). Glyceraldehyde-3-phosphate dehydrogenase (GAPDH) and hypoxanthine-guanine phosphoribosyltransferase (HPRT) were used as housekeeping genes.

qRT-PCR was performed using, PerfeCTa SYBR Green FastMix (Quantabio, Beverly, MA, United States, # 95071) (Ferreira et al., 2022). The primers (Eurofins Genomics, Ebersberg, Germany) used are listed in **Table 1** below. qRT-PCRs was performed on the PCR MyIQ™ 2 Cycler (Biorad, Kabelsketal, Germany), with the IQ 5 software (version: 2.1.97.1001) with triplicate cDNA samples under the following conditions: 95 °C for 30 sec, followed by 45 cycles at 95 °C for 5 seconds, 58 °C for 15 seconds, and extension at 68 °C for 10 min (Ferreira et al., 2022).

**Table 1:** Primers used for the quantitative polymerase chain reaction (qPCR) method.

Primers	Sequence (5’-3’)	Length of cDNA product	Transcript number in Ensembl
GAPDHs GAPDHas	AACTTTGGCATCGTGGAAGGGCT ACCAGTGGATGCAGGGATGATGT	138 bp	ENSRNOT00000050443
HPRTs HPRTas	TTTCCTTGGTCAAGCAGTACAGCCC TGGCCTGTATCCAACACTTCGAGA	89 bp	ENSRNOT00000045153
IL-6s IL-6as	GACAAAGCCAG GTCATTCAGAG TTGGATGGTCTTGGTCCTTAGCC	165 bp	ENSRNOG00000010278
AREGs AREGas	AAGAATCCGTGGCCGCCAAGTTT TTTCTCCACACCGTTCGCCAAAGT	124 bp	ENSRNOG0000002754
MIP-2s MIP-2as	AGAACATCCAGAGCTTGACGGTG GGGCTTCAGGGTTGAGACAAACT	108 bp	ENSRNOG00000002792
MCP-1s MCP-1as	ATGATCCCAATGAGTCGGCTGGAG GCACAGATCTCTCTCTTGAGCTTG	104 bp	NSRNOT00000007159

### 2.7 Cytokine and chemokine determination

Cell culture supernatants were centrifuged at 1000 g for 5 min to remove the cell debris. Concentration of IL-6, IL-10, TNF-alpha, MCP-1, and MIP-2 protein levels from the cell culture supernatants was quantified by available commercial enzyme-linked immunosorbent assay (ELISA) kits (Thermo Scientific; Invitrogen, Darmstadt, Germany) according to the manufacturer´s instructions.

### 2.8 Glucose uptake 2-NBDG assay

Glucose uptake assay was carried out using the 2-NBDG glucose uptake assay kit (K682–50 BioVision, CA, USA). A negative control was prepared with cells treated with a test compound or a control vehicle (Phloretin 1x) with 400 μl cell culture medium with 0.5% FBS at a period of 8-12h. Then, cells were incubated at 37 °C with 6.5% CO_2_ for 1 h. Glucose uptake mix was prepared with 376 μl of cell culture medium (0.5% FBS), 4 μl of 2-NBDG reagent, and 20 μl of glucose uptake enhancer and, was later added to the cells. Cells were incubated for 30 min at 37°C with 6.5% CO_2_ and later kept on ice.

### 2.9 Immunostaining and microscopy

To examine the effects of glucose uptake in AECs L2 subjected to 30% cyclic stretching, cells were washed with PBS (2 x 3 min). Cells were washed twice with ice-cold PBS and fixed with 4% paraformaldehyde (PFA) on ice for 5 min. PFA was washed away twice with PBS (2 x 3 min), and cells were permeabilized with ice-cold 0.1% (v/v) Triton X-100 at room temperature for 5 min. Then, cells were blocked with 3% (w/v) bovine serum albumin source in PBS (20–30 min, RT). Afterward, cells were washed with PBS (2 x 3 min), and incubated at room temperature with 4’,6-diamidino-2-phenylindole (DAPI, 1:200, Merck Sigma-Aldrich, # D-9542) at room temperature for 30 min. The stained cells attached to the silicone membranes were cut off the plates and mounted inverted on slides using the anti-fade mounting reagent (MOWIOL, Calbiochem, # 475904).

Cells were imaged using an Olympus SD-OSR Spinning Disc Confocal Microscope equipped with a 60x numerical aperture N.A. = 1.4 oil DIC objective (Carl Zeiss Microimaging, Jena, Germany) at the Imaging Facility Medical Theoretical Center (MTZ), at University Technical Dresden. Z-stacks were acquired with 1 µm or 3µm intervals between consecutive focal planes. Images were analyzed, processed, and quantified with ImageJ (National Institutes of Health, USA) and Adobe Acrobat Photoshop^®^ 2022 (Adobe, USA. The 3D renditions were obtained from 1µm Z-sections using the ImageJ 3D Viewer plugin (volume view) (Rasband W. S., 2021; Schneider et al., 2012; Schindelin et al., 2015).

### 2.10 Statistical analysis

Data are presented as mean ± standard deviation (SD) from at least three or four independent experiments. Comparisons among the groups were conducted with a one-way ANOVA for the factors condition (non-Stretch, LPS, Stretch, and LPS+Stretch) followed by a Šidák post-test. Analyses were conducted using GraphPad Prism version 10.4.1 (GraphPad Software Inc., USA). Statistical significance was defined as *p* < 0.05.

Correlation between glucose uptake and inflammatory markers was assessed using condition medians for Control, Stretch, LPS, and LPS+Stretch groups at 1 h and 4 hrs. Analyses were organized into predefined blocks including No LPS (Control + Stretch), +LPS (LPS + LPS+Stretch), Stretch vs Control, Stretch only, and all conditions pooled. Correlations were calculated between glucose uptake and inflammatory markers measured by RT-qPCR (AREG, IL-6, MCP-1, and MIP-2) and ELISA (IL-6, IL-10, TNF-α, MCP-1, and MIP-2). Both Pearson and Spearman correlation coefficients were computed. Because several block comparisons contained small sample sizes, exact two-sided permutation tests were used to calculate *p values.* Correlations were considered statistically significant at *p < 0.05.* Correlation analyses were performed using Python 3.11 with NumPy 1.26, Pandas 2.x, SciPy 1.11, Matplotlib 3.8, and OpenPyXL 3.1.

## 3 Results

### 3.1 Cell viability under 30% stretch cells

Exposure of L2 alveolar epithelial cells to 30% cyclic stretch for 1 or 4 hours did not affect cell viability, as assessed by lactate dehydrogenase (LDH) release. (**Figure 1**).

**Figure 1:**
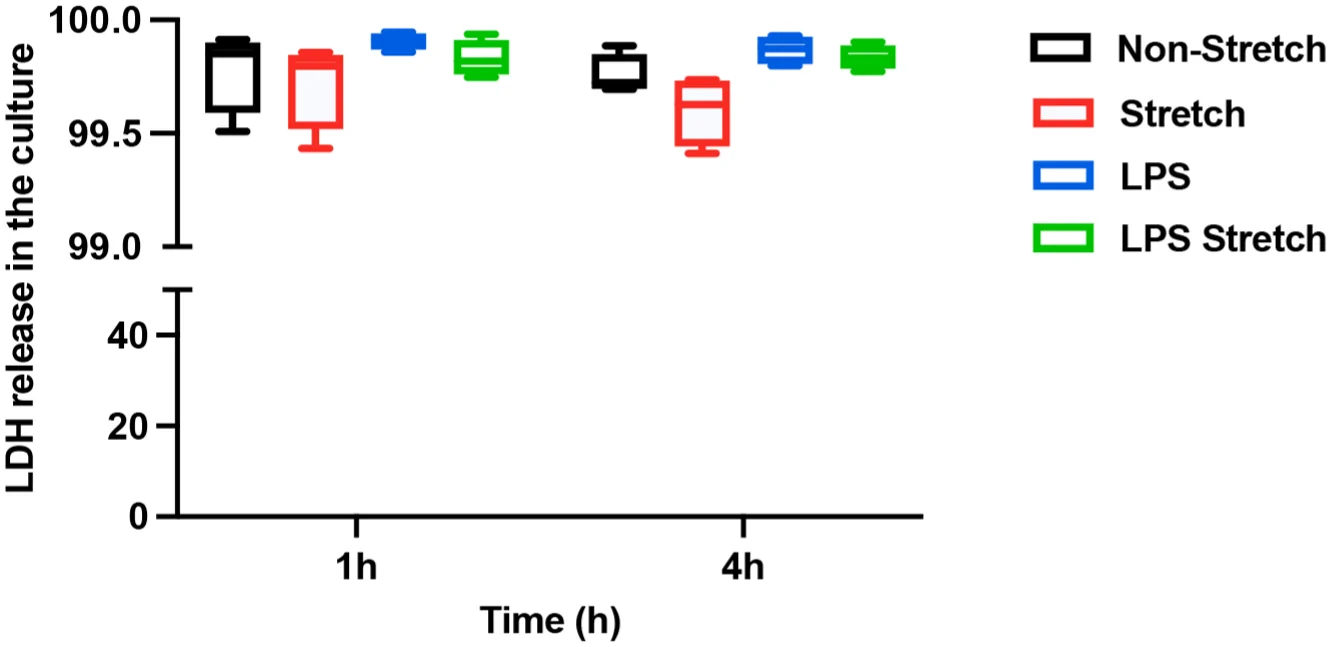
Cell viability in alveolar epithelial cells type II. Cells were exposed to 30% cyclic stretch for 1 h and 4 hours.

### 3.2 Effect of glucose oxidase activity under stretched cells

As shown in **Figure 2**, exposure to 30% cyclic stretch for 1 and 4 hours significantly increased glucose oxidase activity in L2 AECs, both in the presence and absence of lipopolysaccharide, compared with non-stretched controls. Data are presented as n = 4 per group.

**Figure 2:**
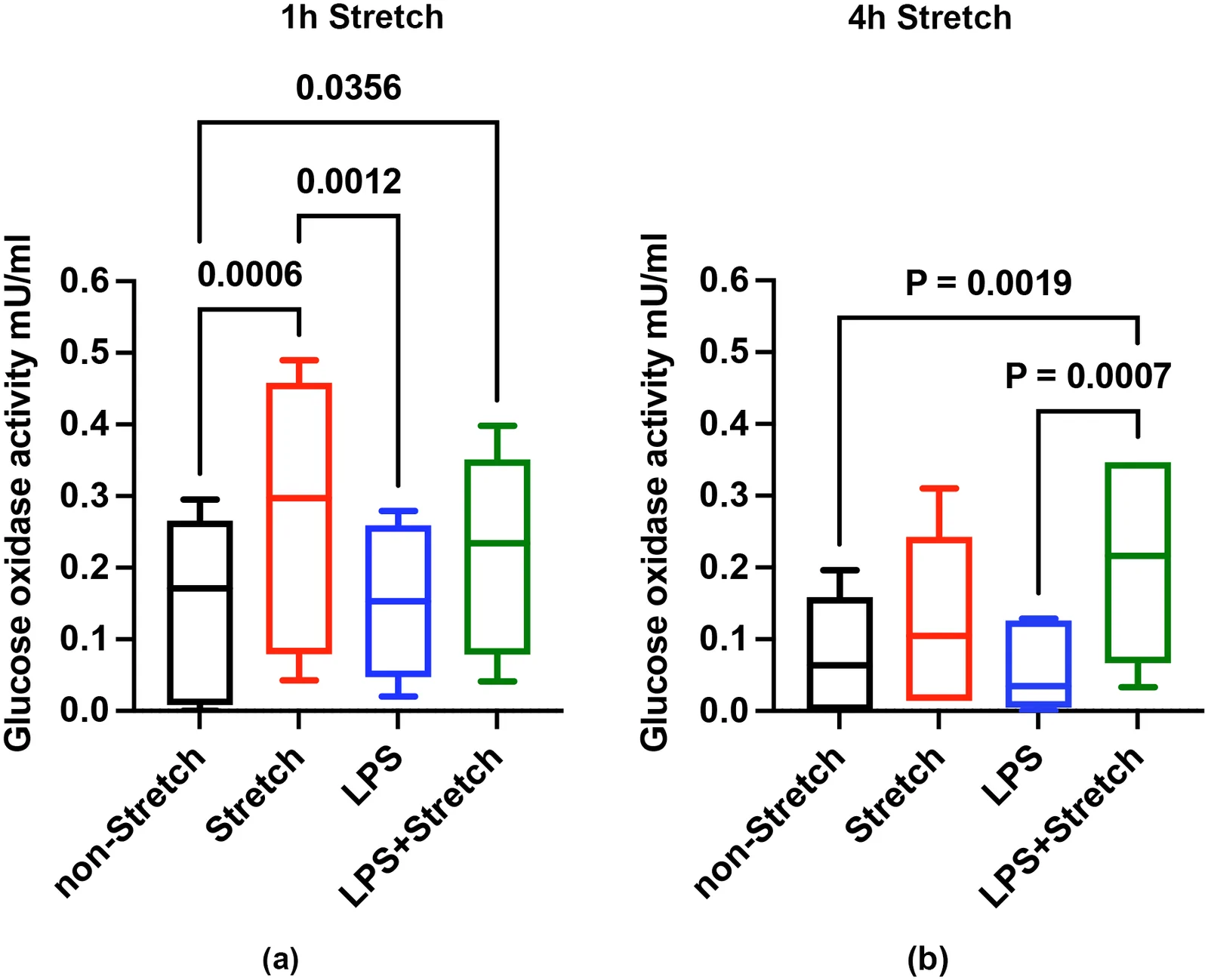
Effects of 30% cyclic stretch on the glucose oxidase metabolism in treated and untreated alveolar epithelial type II cells with LPS at 1 and 4 hours. L2 AECs were treated and untreated with lipopolysaccharide (LPS, 2µg/ml), and exposed or not in a cyclic stretch condition of 30% at 1 h **(A)** and 4 h **(B)**. Non-LPS and un-stretch cells (control) are shown with a black color, non-LPS and stretch cells are shown with a red color, LPS, and un-stretch cells are shown in a blue color, and LPS and stretch cells are shown with a green color in the graph respectively. Statistical significance between control and stretch, as well as LPS and non-LPS with or without stretch, was at *p* < 0.05 and *p* < 0.0001. Data are presented as n = 4 per group.

In cells under an excessive 30% stretch without LPS, glucose oxidase levels increased at 1 h (*p* = 0.0356) and 4 h (*p* = 0.0019) compared with non-stretched, non-LPS controls. In addition, LPS-treated cells under 30% stretch for 4 h showed higher levels (*p =* 0.0007) than LPS-treated, non-stretched cells.

### 3.3 Gene expression in L2 cells under stretch 30% and pro-inflammatory cytokines

LPS-stimulated L2 AECs under stretch, AREG expression increased significantly (*p* = 0.0004) at 1 h and 4 hours (*p* = 0.0154) compared with cells treated with LPS and non-stretched, **Figures 2A, E**.

At 1 h, IL-6 and MIP-2 expression were also higher in stretched LPS-treated cells than in LPS-treated non-stretched controls (*p* = 0.00295 and *p* = 0.00453, respectively; **Figures 3B–D**) or in cells without LPS non-stretched controls (p = 0.0030 and *p =* 0.0083). At 4 h, IL-6, MCP-1, and MIP-2 were significantly increased in stretched LPS-treated cells compared with non-stretched, non-LPS cells (*p* = 0.0074, *p* = 0.0378, and *p* < 0.0001, respectively; **Figures 3E–H**).

**Figure 3:**
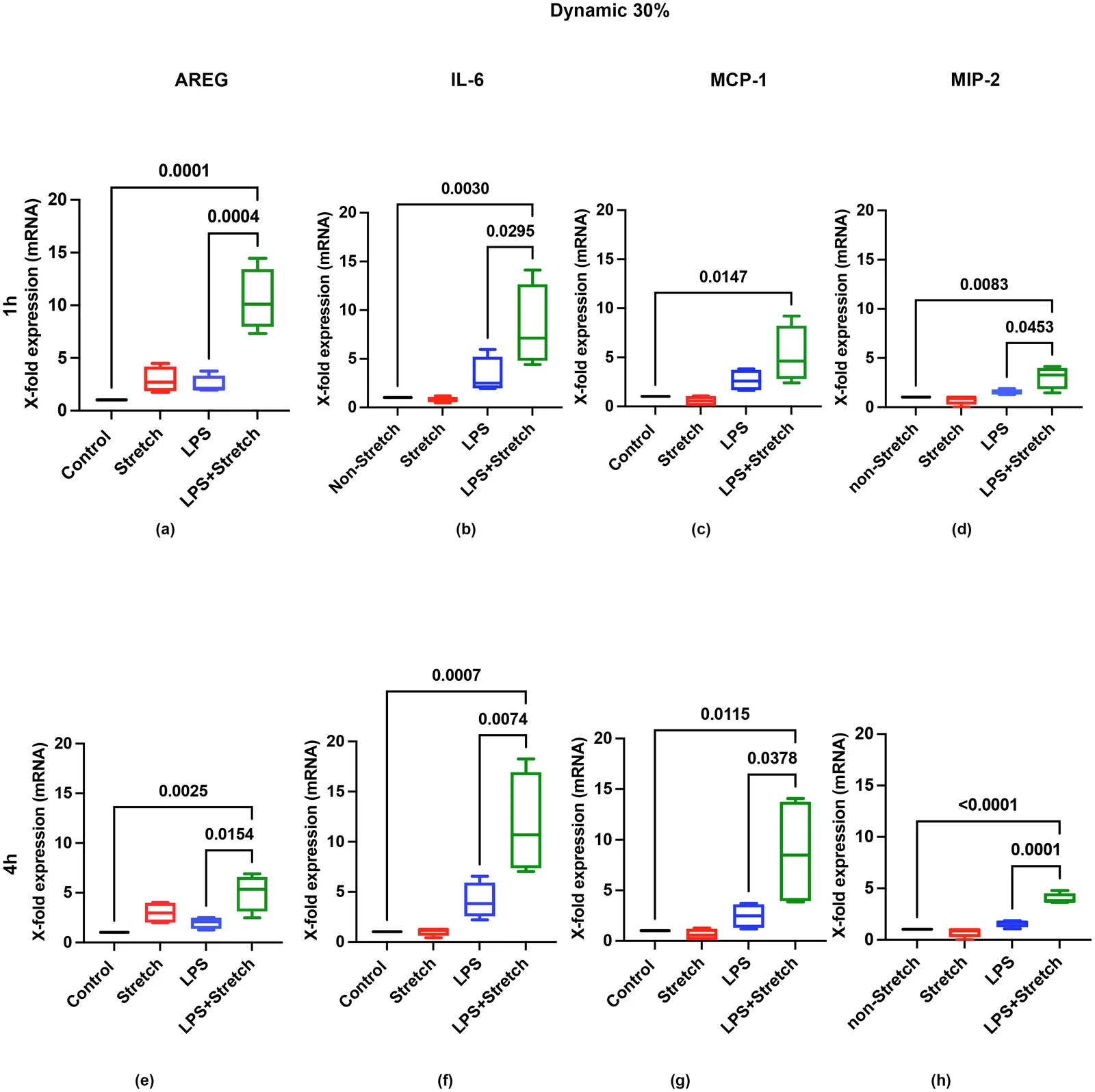
Effects of 30% cyclic stretch in alveolar epithelial type II cells treated and untreated with LPS on the gene expression of AREG, IL-6, MCP-1, and MIP-2 at times 1 and 4 hours. L2 AECs were treated with lipopolysaccharide (LPS, 2µg/ml), and exposed to a non-stretch and stretch of a dynamic stretch of 30%. RNA was isolated, reverse transcribed, and the cDNA products for **(A)** AREG, **(B)** IL-6, **(C)** MCP-1, and **(D)** MIP-2 for 1 h and **(E)** AREG, **(F)** IL-6, **(G)** MCP-1, and **(H)** MIP-2 for 4 h and were analyzed by semiquantitative RT-PCR using the ΔΔCT method. Data presented is the X-fold expression (mRNA) compared to non-stretched cells treated and untreated with LPS (n=3 per group). Non-LPS and unstretched cells (control) are shown with a black color, non-LPS and stretched cells are shown with a red color, unstretched and treated LPS cells are shown in a blue color stretched and treated LPS cells are shown with a green color in the graph respectively. Statistical significance between control and stretch, LPS and non-LPS at a dynamic stretch of 30% was at *p* < 0.05 and *p* < 0.0001.

### 3.4 Protein expression during the stretch

LPS-treated L2 AECs exposed to 30% stretch for 1 h showed significantly increased secretion of IL-6, MCP-1, and MIP-2 (*p* = 0.0103, *p* = 0.0057, and *p* = 0.0449, respectively) compared with LPS-treated non-stretched controls. In contrast, no significant changes in IL-10 or TNF-α were observed in cells with or without LPS under 30% stretch at 1 h (**Figures 4A–E**).

**Figure 4:**
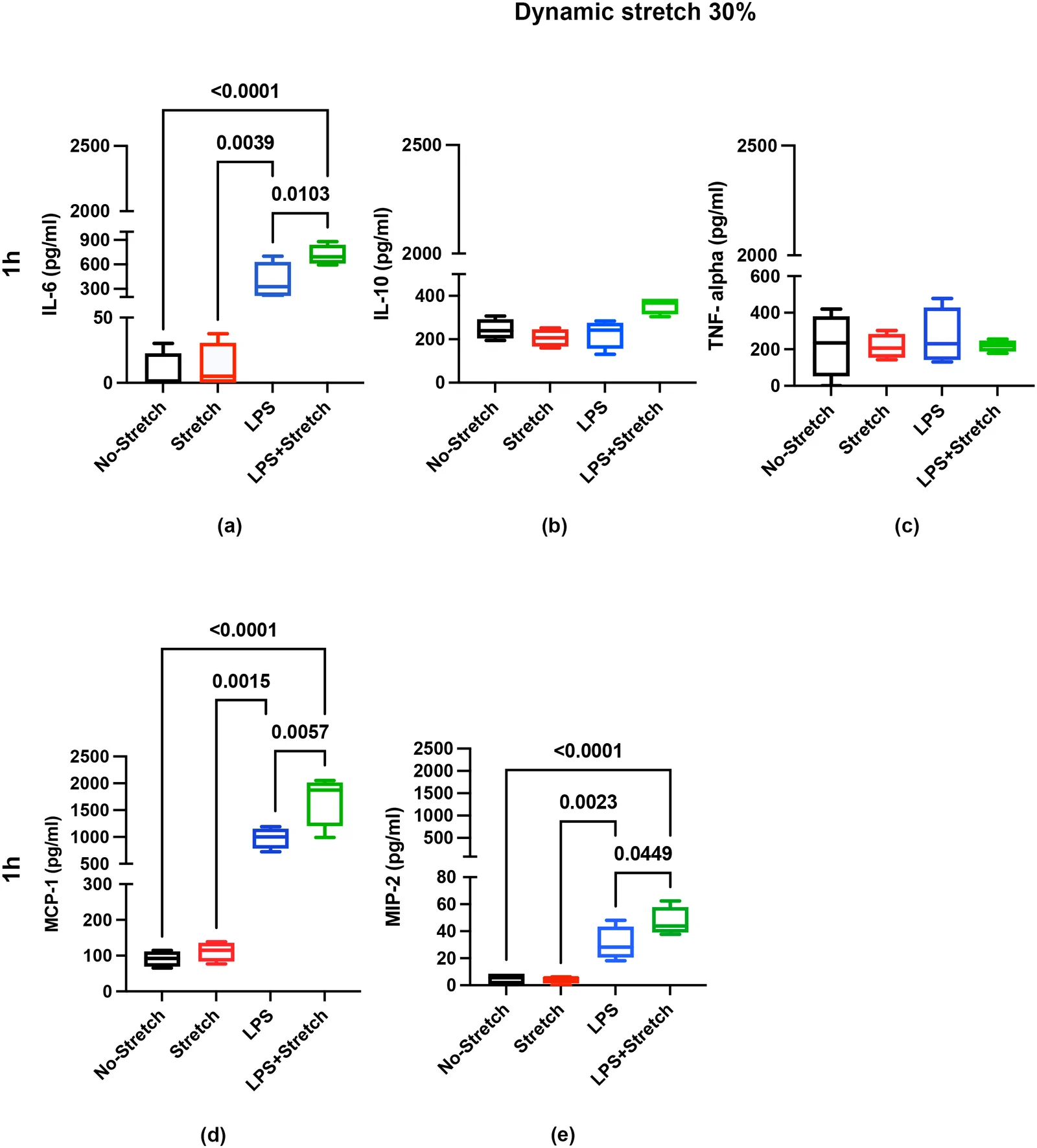
Effect of 30% cyclic stretch on the secretion of IL-6, IL-10, TNF-alpha, MCP-1, and MIP-2 in alveolar epithelial cells type II at 1 hour. L2 AECs were untreated and treated with lipopolysaccharide (LPS, 2µg/ml) and exposed to non-stretched and stretched conditions for 1h. Cell culture supernatants were analyzed for **(A)** IL-6, **(B)** IL-10, **(C)** TNF-alpha, **(D)** MCP-1, and **(E)** MIP-2 by ELISA Kits (n=3 per group). Non-LPS and unstretched cells (control) are shown with a black color, non-LPS and stretched cells are shown with a red color, unstretched and treated LPS cells are shown in a blue color stretched and treated LPS cells are shown with a green color in the graph, respectively. Statistical significance between control and stretch, LPS and non-LPS under a cyclic stretch of 30% was at *p* < 0.05 and *p* < 0.0001.

At 4 hours, secretion of IL-6, TNF-α, and MCP-1 did not differ significantly between stretched and non-stretched cells, with or without LPS (**Figures 5A, C**). In contrast, IL-10, MCP-1, and MIP-2 were significantly increased in LPS-stimulated cells exposed to cyclic stretch only compared with non-stretched, non-LPS controls (*p* = 0.0359, *p* = 0.0030, and p = 0.0014, respectively; **Figures 5B, D, E**).

**Figure 5:**
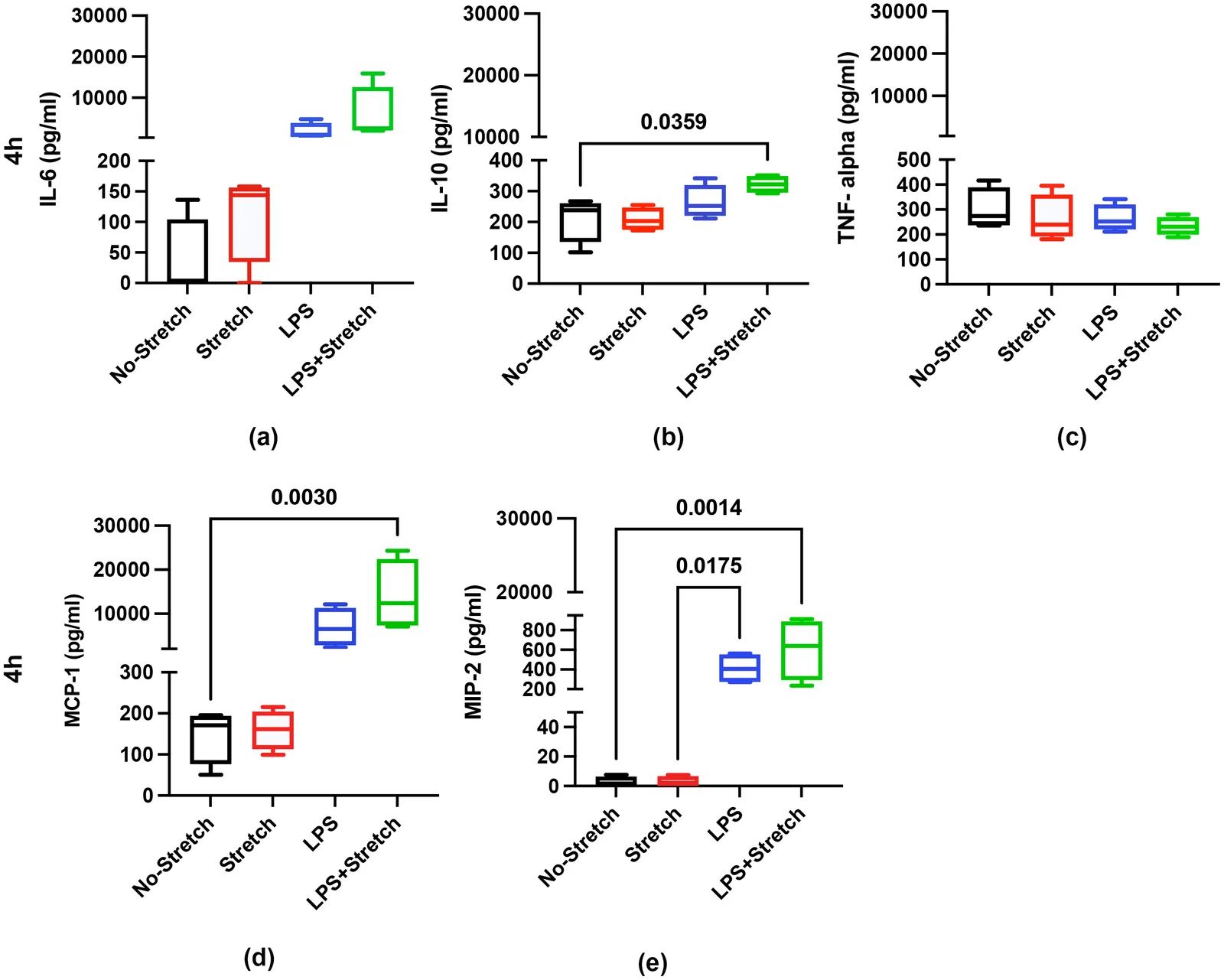
Effect of 30% cyclic stretch on the secretion of IL-6, IL-10, TNF-alpha, MCP-1, and MIP-2 in alveolar epithelial type II cells at 4 hours. L2 AECs were untreated and treated with lipopolysaccharide (LPS, 2µg/ml) and exposed to non-stretch and stretch in the period 4 hours. Cell culture supernatants were analyzed for **(A)** IL-6, **(B)** IL-10, **(C)** TNF-alpha, **(D)** MCP-1, and **(E)** MIP-2 by ELISA Kits (n=3 per group). Non-LPS and un-stretch cells (control) are shown with a black color, non-LPS and stretch cells are shown with a red color, un-stretch and treated LPS cells are shown in a blue color stretch and treated LPS cells are shown with a green color in the graph, respectively. Statistical significance between control and stretch, LPS and non-LPS at a dynamic stretch of 30% was at *p* < 0.05 and *p* < 0.0001.

### 3.5 Effects of 2-NBDG uptake in alveolar epithelial type II cells under stretch

Fluorescence microscopy revealed that uptake of the fluorescent glucose analog 2-NBDG was predominantly localized to the perinuclear region of alveolar epithelial type II cells. 2-NBDG uptake is increased in AECs type II cells with and without LPS under 30% stretch at 1 h (**Figure 6**) and 4 hours (**Figure 7**).

**Figure 6:**
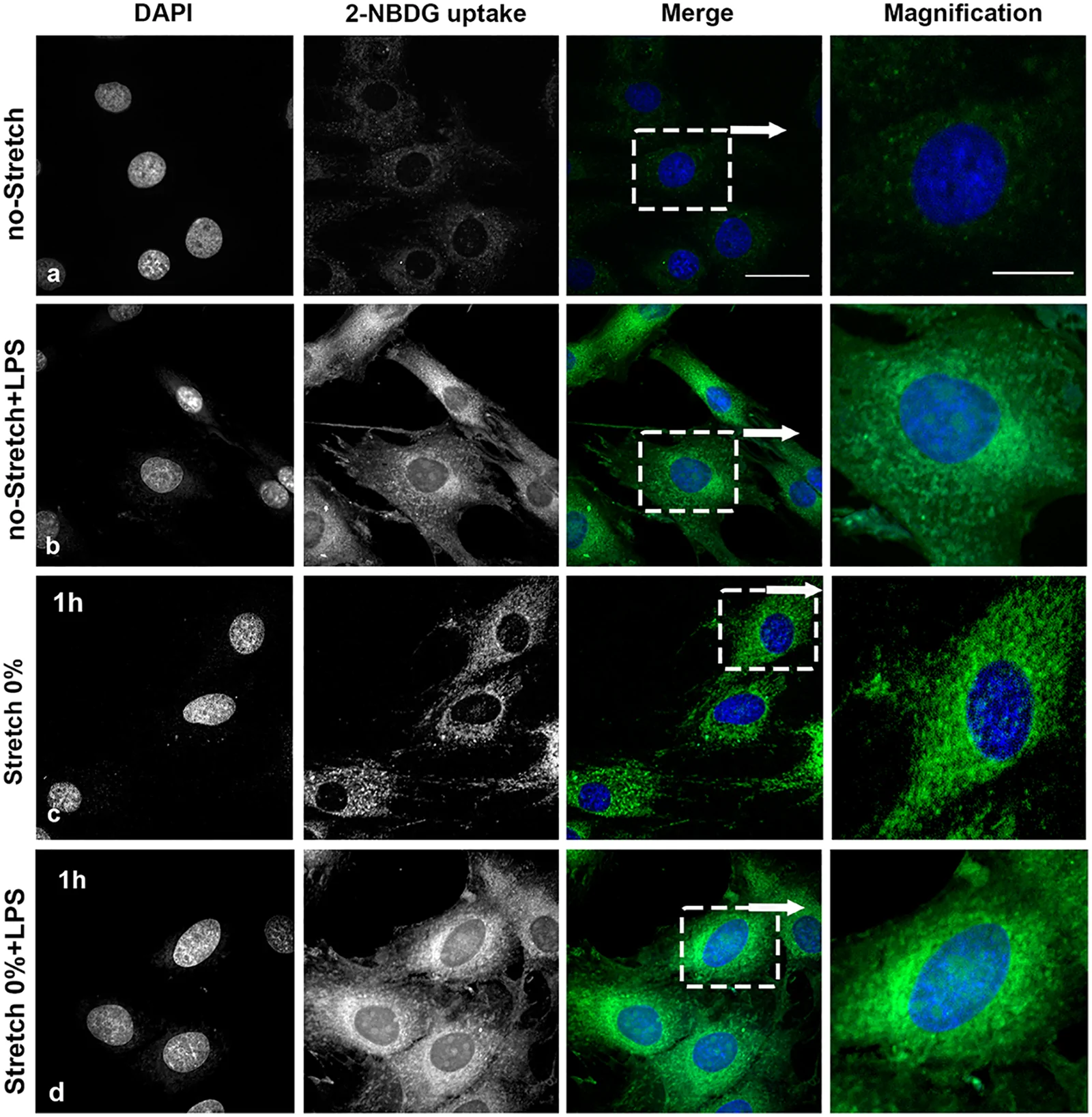
Effects of 30% cyclic stretch on the 2-NBDG uptake at time 1 h in alveolar epithelial cells type II with and without LPS. L2 AECs were treated or not with lipopolysaccharide (LPS, 2µg/ml). Arrows show the boxed areas of 2-NBDG [2-(N-(7-Nitrobenz-2-oxa-1,3-diazol-4-yl)amino)-2-deoxyglucose], a fluorescently tagged glucose tracer assay, from the perinuclear region in AECs II undergoing 30% cyclic stretch for 1 h. **(A)** non-Stretch, **(B)** non-Stretch+LPS, **(C)** stretch, and **(D)** Stretch+LPS. Cells were stained, fixed, and analyzed by confocal microscopy. Single channels are displayed in greyscale for DAPI (DNA) and 2-NBDG; Merge and Magnification: 2-NBDG (green) and DAPI (blue). Data are displayed as projections of 1μm Z-sections (n=4). Scale bars: 30μm.

**Figure 7:**
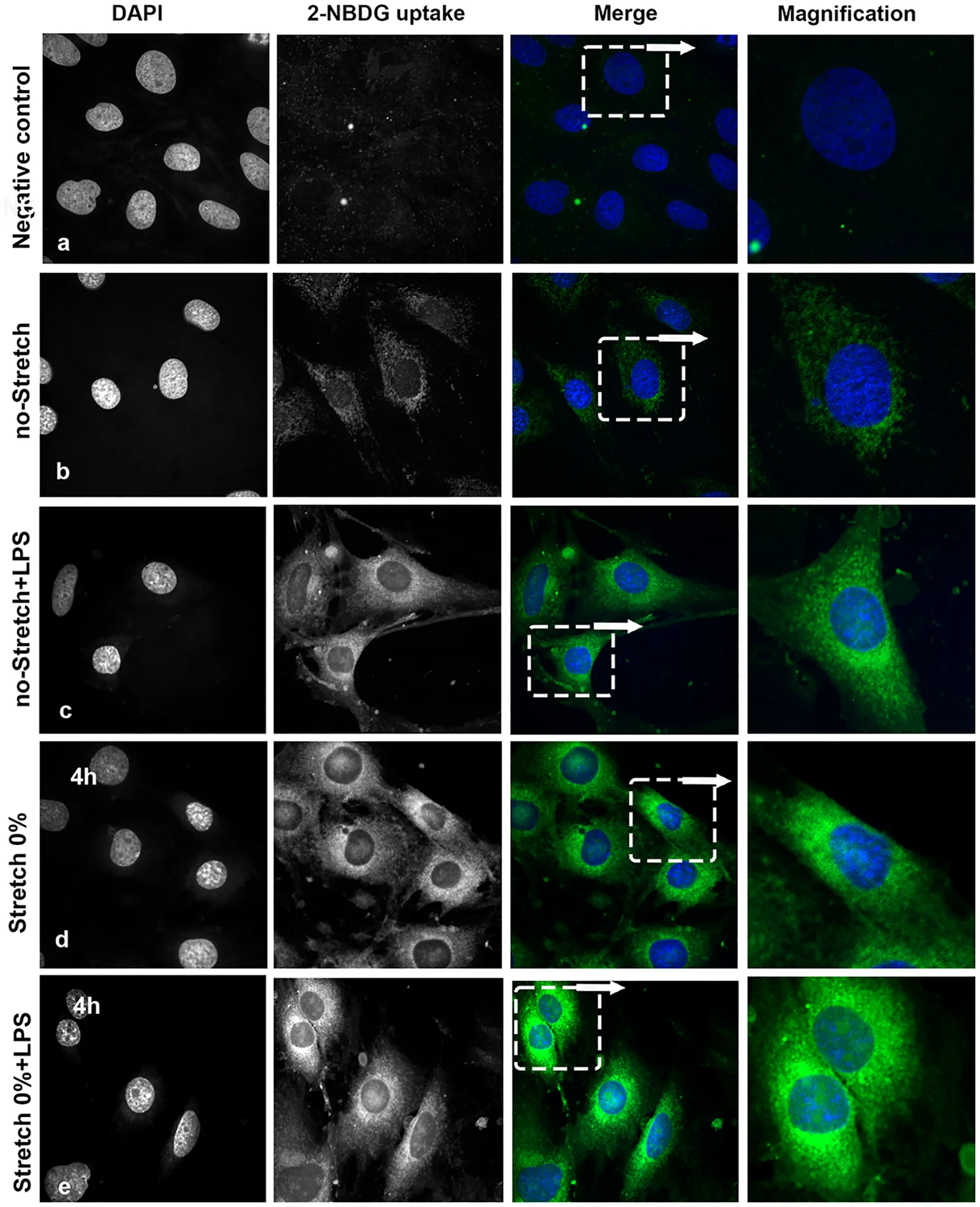
Effects of 30% cyclic stretch on the 2-NBDG uptake at time 4 h in the alveolar epithelial cells type II with and without LPS. L2 AECs were treated or not with lipopolysaccharide (LPS, 2µg/ml). Arrows show the boxed areas of 2-NBDG [2-(N-(7-Nitrobenz-2-oxa-1,3-diazol-4-yl)amino)-2-deoxyglucose], a fluorescently tagged glucose tracer assay, from the perinuclear region in L2 AECs undergoing 30% cyclic stretch at 4 hours. **(A)** Ploretin (negative control), **(B)** non-Stretch, **(C)** non-Stretch+LPS, **(D)** stretch, and **(E)** Stretch+LPS. Cells were stained, fixed, and analyzed by confocal microscopy. Single channels are displayed in grey scale for DAPI (DNA) and 2-NBDG; Merge and Magnification: 2-NBDG (green) and DAPI (blue). Data are shown as projections of 1μm Z-sections (n=4). Scale bars: 30 μm.

### 3.6 NBDG uptake fluorescence intensity

The fluorescence intensity of 2-NBDG uptake was significantly higher in L2 AECs, with or without LPS, under 30% stretch at both 1 and 4 hours. Stretched cells, treated or untreated with LPS, showed increased uptake at 1 h and 4 hours (both *p* < 0.0001) compared with non-stretched, non-stimulated cells or non-stretched LPS-treated cells. Moreover, LPS-stimulated stretched L2 AECs exhibited higher fluorescence at 1 h (*p* = 0.0014*)* and 4 hours (*p* < 0.0001) than non-stretched, non-LPS controls. These findings indicate that excessive mechanical stretch enhances glucose uptake in L2 AECs (**Figure 8**).

**Figure 8:**
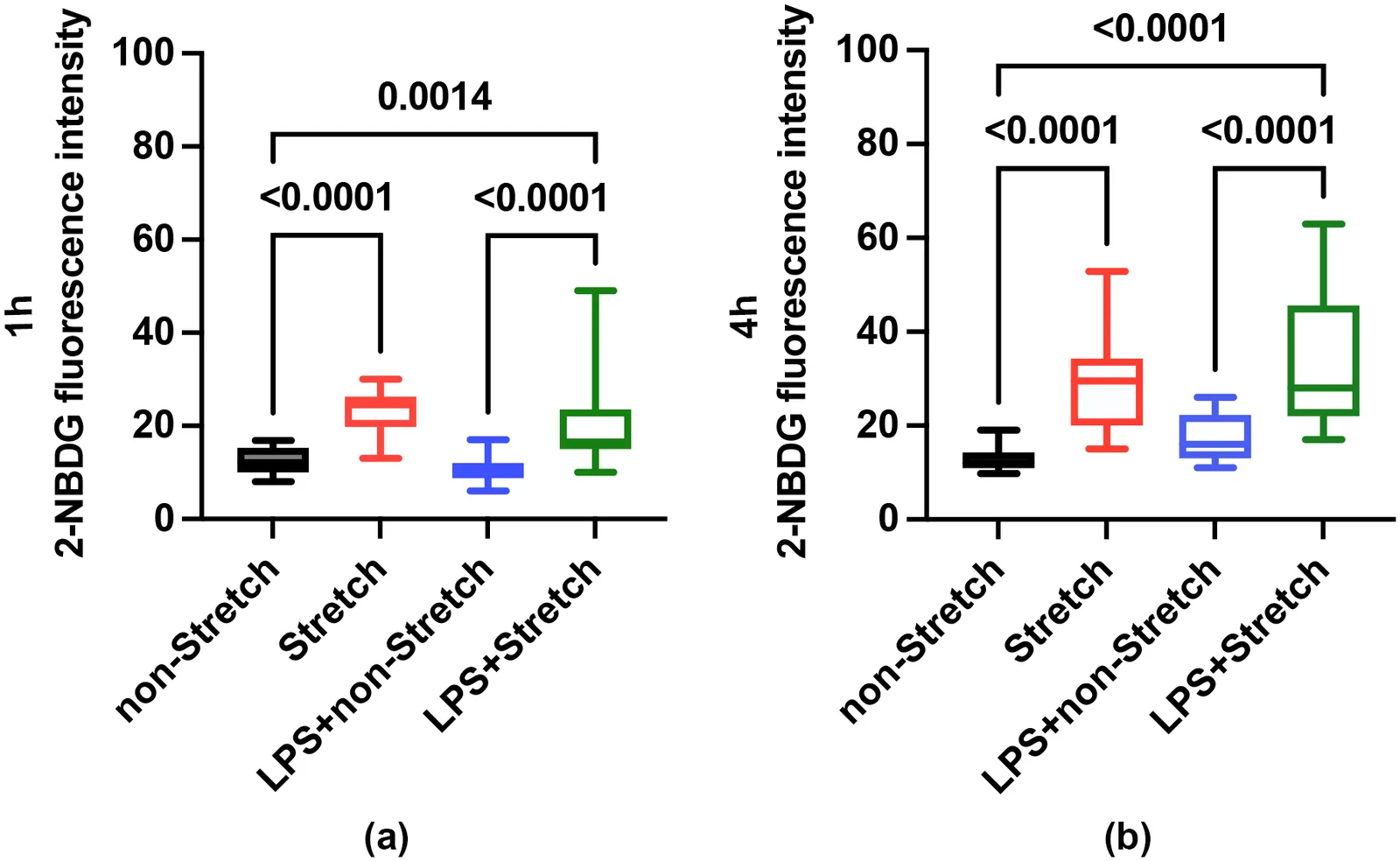
Fluorescence intensity quantification of the 2 -NBDG marker in the alveolar epithelial type II cells with and without LPS under a stretch of 30% at 1 and 4 hours. **(A)** Cells were treated and non-treated with LPS and stretched or not at 1 h, **(B)** Cells were treated and non-treated with LPS and stretched or not at 4 hours. Data are presented as mean±SD (n=4). Differences were considered statistically significant at *p* < 0.05 and *p <*0.0001.

### 3.7 Correlation between glucose uptake and inflammatory markers

To explore the relationship between metabolic activation and inflammatory signaling, in our experimental model, exploratory correlation analyses were performed between glucose uptake and inflammatory markers measured by RT-qPCR and ELISA at 1 and 4 hours. Correlation analyses were not performed using individual biological replicates. Instead, for each experimental condition, the median value obtained from the independent biological experiments (n = 3–4 per condition) was calculated and used as a single representative observation. Correlation tables were generated using these condition-level median values for the Control, Stretch, LPS, and LPS+Stretch groups. Analyses were organized into predefined blocks, including No LPS, +LPS, Stretch vs Control, Stretch only, and all conditions pooled.

Consequently, the number of observations (N) reported in the correlation tables corresponds to the number of condition-level median values included in each correlation block and should not be interpreted as the number of independent biological experiments. Because some blocks contained fewer than three, correlations for these conditions were considered not estimable and were reported as N.E. Exact two-sided permutation tests were used to calculate p values. Both Pearson and Spearman correlation coefficients were computed **Supplementary Figure 2**.

## 4 Discussion

The main findings of this study demonstrate that exposure of rat alveolar epithelial type II cells to excessive cyclic stretch (30%) for 1 or 4 hours, with LPS stimulation, significantly increased both 2-NBDG uptake and glucose oxidase activity, compared with non-stretched control cells, with or without LPS treatment. These two measurements represent distinct glucose-related experimental readouts and should not be interpreted interchangeably. In contrast, the inflammatory response was predominantly observed in LPS-treated cells and varied according to the inflammatory mediator and exposure time. Thus, the effects of cyclic stretch on the glucose-related readouts assessed in this study were more consistent than its effects on inflammatory signaling.

To trigger the inflammatory response in L2 AECs we used LPS, which is widely employed to induce inflammation *in vitro* and *in vivo* (Dreyfuss et al., 1988; McRitchie et al., 2000; Birukova et al., 2012; Wong and Johnson, 2013) or to model ARDS (Pugin et al., 1998; Ding et al., 2013). Stretch conditions were performed in L2 AECs because they can be easily grown under controlled conditions and are the main cell type involved in the immune response of the lung alveolar epithelium (Kang and Kim, 2017).

It is well known that injured lungs are at risk of further deterioration due to overstretching during mechanical ventilation (Marini and Gattinoni, 2020).

In ARDS, tidal volumes >8 mL/kg are associated with alveolar overstretch (volutrauma), which causes significant lung injury and increased mortality (Terragni et al., 2003; Pellegrini et al., 2024). Accordingly, our data show that 30% cyclic stretch increased both 2-NBDG uptake and glucose oxidase activity in LPS-treated and untreated cells. Importantly, these measurements provide complementary but distinct information. 2-NBDG is a fluorescent glucose analogue and its intracellular fluorescence primarily reflects cellular glucose analogue uptake, whereas the glucose oxidase assay represents a separate enzymatic readout measured in cell lysates. Therefore, the parallel increase in these parameters should not be interpreted as evidence that they represent the same biological process or that one directly causes the other.

As described in the literature, positron emission tomography/computed tomography (PET/CT) imaging of 18F-FDG is a valuable method to determine the pulmonary inflammatory response in ARDS patients (Prost et al., 2010; Pauwels et al., 2013; Braune et al., 2019). The radiolabeled ^18^F-FDG is transported into cells via the same GLUT family of transporter proteins as glucose (primarily GLUT-1). In the presence of hexokinase, it is phosphorylated to ^18^ F-FDG-6-phosphate. Unlike glucose-6-phosphate, this metabolite cannot undergo further metabolism via the Krebs cycle, leading to intracellular accumulation that is proportional to cellular glucose uptake and hexokinase activity (Wang et al., 2017; Cruz et al., 2018).

Importantly, pulmonary 18F-FDG uptake measured *in vivo* reflects the combined metabolic activity of multiple pulmonary cell populations, including neutrophils, macrophages, endothelial cells and epithelial cells (Crosby et al., 2011). Therefore, the present findings should be interpreted within the context of alveolar epithelial cells and should not be directly extrapolated to whole-lung inflammatory activity.

On the cellular level, our results show increased perinuclear localization and fluorescence intensity of 2-NBDG under cyclic stretch, consistent with increased uptake of the fluorescent glucose analog. However, because glucose transporter expression or activity was not directly measured, these findings cannot establish whether the increased 2-NBDG signal results from altered GLUT expression, transporter activity, or other mechanisms. Further studies assessing glucose transporters such as GLUT1 and GLUT3 together with downstream metabolic pathways will be required to distinguish between these possibilities.

LPS stimulation markedly enhanced inflammatory mediator expression and secretion, whereas the additional effect of cyclic stretch varied among cytokines and time points. The increased gene expression and release of IL-6, MCP-1, and MIP-2 by LPS-stimulated AECs L2 is consistent with previous studies reporting stretch-induced inflammatory signaling in alveolar epithelial cells (Dreyfuss and Saumon, 1998; Suki and Hubmayr, 2014; Tanaka et al., 2017; Bates and Smith, 2018; Pelosi et al., 2018; Ferreira et al., 2022). Amphiregulin is involved in epithelial repair and inflammation, acting as a growth factor and receptor ligand. From our findings, an increase in AREG expression at 1 or 4 hours during mechanical ventilation is consistent with published studies reporting elevated AREG levels in ventilated adult mice (Hillman et al., 2014).

A key strength of this study is the use of a controlled *in vitro* stretching model that reproduces stretch magnitudes comparable to those experienced during injurious mechanical ventilation. Although 30% cyclic stretch exceeds physiological alveolar deformation, it has been widely used as an experimental surrogate of severe alveolar overdistension associated with ventilator-induced lung injury. Thus, a customized stretching device was used that mimics *in vivo* mechanical stress during MV, as mechanotransduction is critical in VILI (Chen et al., 2018). The selected stretch duration (1 and 4 hours) allowed detection of early metabolic and inflammatory responses. However, it cannot fully recapitulate the complexity of the *in vivo* alveolar environment. If the lung volume increases by 40–100% of the total lung capacity, the basal surface area of alveolar epithelial cells increases by 34–35% (Tschumperlin and Margulies, 1999; Tschumperlin et al., 2000; Wirtz and Dobbs, 2000).

Given the limitations of current rodent models of mechanical ventilation to short time frames and the rapid cellular responses of inflammation and glucose uptake, our experiments were conducted at 1 and 4 hours. Previous studies have demonstrated that the overdistension stretch model effectively induces cellular deformation (Ferreira et al., 2022; Ran et al., 2023).

Our previous studies have shown that excessive 30% cyclic stretch induces cytoskeletal remodeling in L2 AECs, including F-actin stress fiber formation and increased paracellular permeability (Ferreira et al., 2022; Ran et al., 2023). The increased glucose uptake observed in the present study may therefore reflect the energetic requirements associated with cytoskeletal remodeling, maintenance of epithelial integrity, transcriptional activation, and protein synthesis. However, the molecular pathways responsible for this metabolic response were not directly investigated. Importantly, the molecular mechanisms responsible for the increased 2-NBDG uptake and glucose oxidase activity were not investigated in the present study. Therefore, our data cannot determine whether cyclic stretch alters glucose transporter expression or activity, intracellular glucose metabolism, glucose oxidase-related enzymatic activity, or a combination of these processes. Based on mechanisms described in the literature, future studies could investigate whether glucose transporters, such as GLUT1/GLUT3, mechanotransduction pathways such as ERK1/2 and YAP, or metabolic regulators including AMPK, HIF-1α, and PI3K/Akt/mTOR signaling (Ouiddir et al., 1999; Correa-Meyer et al., 2002; Nam et al., 2026). Future studies are warranted to investigate these pathways and establish the molecular link between mechanical stress, glucose metabolism, and inflammatory activation.

Our findings show that, in rat L2 AECs, the glucose-related and inflammatory responses exhibited different patterns across the experimental conditions and time points. Importantly, within the limitations of the present *in vitro* epithelial model, increased 2-NBDG uptake was not consistently accompanied by increased inflammatory mediator expression or secretion. However, the present study was not designed or powered to establish whether glucose uptake can serve as a surrogate marker of inflammatory activation. Furthermore, these observations should not be extrapolated to the *in vivo* lung, where glucose uptake reflects the combined metabolic activity of multiple cell populations, including neutrophils, macrophages, endothelial cells, and epithelial cells.

Exploratory correlation analyses are provided in **Supplementary Figure 2**. Because these analyses were based on a limited number of condition-level median observations, they were not powered to detect meaningful associations and were not considered suitable for inferential conclusions. Accordingly, these analyses should be regarded as hypothesis-generating only and cannot establish or exclude an association between 2-NBDG uptake and inflammatory responses.

Thus, **Figure 9** provides a schematic summary of the principal experimental findings obtained in the present study: excessive 30% cyclic stretch increased 2-NBDG uptake and glucose oxidase activity in rat L2 alveolar epithelial cells, whereas inflammatory responses were more pronounced in LPS-treated cells and varied according to the inflammatory mediator and experimental time point.

**Figure 9:**
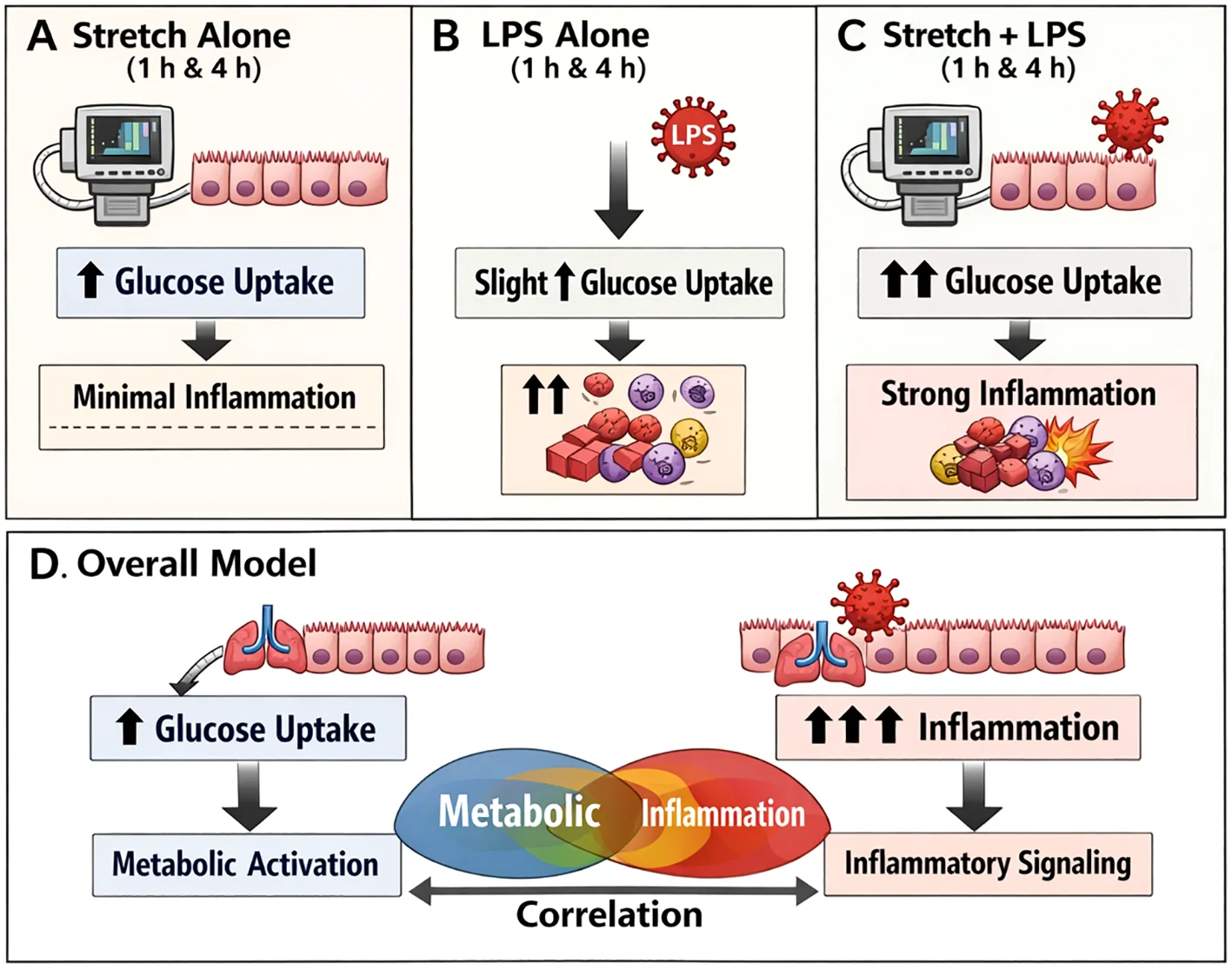
Represents a conceptual summary of the principal findings obtained in the present study. Schematic representation of the principal experimental findings observed following exposure to 30% cyclic stretch and/or lipopolysaccharide (LPS) for 1 h and 4 hours. **(A)** Cyclic stretch alone consistently increased glucose uptake and glucose oxidase activity, indicating metabolic activation in alveolar epithelial cells, while inflammatory responses remained limited in the absence of LPS priming. **(B)** LPS stimulation alone promoted inflammatory activation with comparatively modest effects on glucose uptake. **(C)** Combined cyclic stretch and LPS stimulation resulted in increased glucose uptake together with enhanced inflammatory responses compared with non-stretched control cells, however, the magnitude of the inflammatory response varied among the inflammatory mediators and experimental time points evaluated. **(D)** Overall schematic summary of the experimental findings. Excessive cyclic stretch consistently increased glucose uptake, whereas inflammatory responses were more prominent in LPS-treated cells. The relationship between metabolic activation and inflammatory responses varied according to the inflammatory mediator and experimental time point. This schematic summarizes the observed experimental findings and should not be interpreted as evidence of a direct mechanistic coupling or causal relationship between glucose metabolism and inflammatory signaling.

Overall, our findings demonstrate that excessive cyclic stretch increases two distinct glucose-related readouts, 2-NBDG uptake and glucose oxidase activity, in rat L2 alveolar epithelial cells, whereas inflammatory responses were more strongly influenced by LPS stimulation and varied among mediators and time points. The present study does not establish the molecular mechanisms underlying the observed glucose-related changes or a causal relationship between the two readouts. These observations provide a basis for future mechanistic studies investigating how mechanical deformation influences glucose transport, cellular metabolism, and inflammatory signaling in alveolar epithelial cells.

### 4.1 Possible implications of the findings

The present findings provide insight into the cellular responses of alveolar epithelial cells to excessive mechanical deformation. Excessive ventilation is able to cause lung injury, which can be seen as volutrauma; it can also be explained by biotrauma.

In L2 AECs, 30% cyclic stretch increased two distinct glucose-related readouts, 2-NBDG uptake and glucose oxidase activity, whereas inflammatory responses were more strongly influenced by LPS stimulation and varied among inflammatory mediators and time points. These findings indicate that glucose-related and inflammatory responses may exhibit different patterns following mechanical stress.

Importantly, increased 2-NBDG uptake was not consistently accompanied by increased expression or secretion of inflammatory mediators. Therefore, these observations should be restricted to the present *in vitro* epithelial model and should not be extrapolated to whole-lung inflammatory activity or clinical responses to mechanical ventilation. Further studies using more complex experimental models are required to determine the mechanisms underlying these glucose-related responses and their relationship with inflammatory signaling.

### 4.2 Limitation

Our study holds several limitations. First, *in vitro* conditions cannot fully replicate the complex multicellular and mechanical environment of the lung parenchyma *in vivo.* Second, LPS is a complex inflammatory stimulus that may contain additional bacterial components and exhibits cytotoxic effects; however, it remains a widely accepted and reproducible model for inducing inflammation *in vitro*. Third, the mechanical forces transmitted through a collagen-coated membrane differ from those experienced by epithelial cells *in vivo*, where stretch is applied through interactions with the extracellular matrix and neighboring cells.

### 4.3 Conclusion

In summary, rat alveolar epithelial L2 cells exposed to 30% cyclic overstretch exhibited increased glucose-related responses, whereas inflammatory responses were more strongly influenced by LPS stimulation and varied among mediators and time points. These findings indicate that glucose-related and inflammatory responses exhibit different patterns under excessive mechanical stretch and provide a basis for future mechanistic studies of epithelial responses to mechanical deformation.

## Data Availability

The datasets presented in this study can be found in online repositories. The names of the repository/repositories and accession number(s) can be found in the article/**Supplementary Material**.
