# Supplementary material for: Cyclic stretch increases glucose uptake and pro-inflammatory response in rat alveolar epithelial type II cells
Source: Front Physiol. 2026 Oct 1;17:1909536. doi: 10.3389/fphys.2026.1909536 (PMC13668563; doi:10.3389/fphys.2026.1909536)
Supplement: Supplementary file 1 [file DataSheet1.pdf]

Supplementary Material

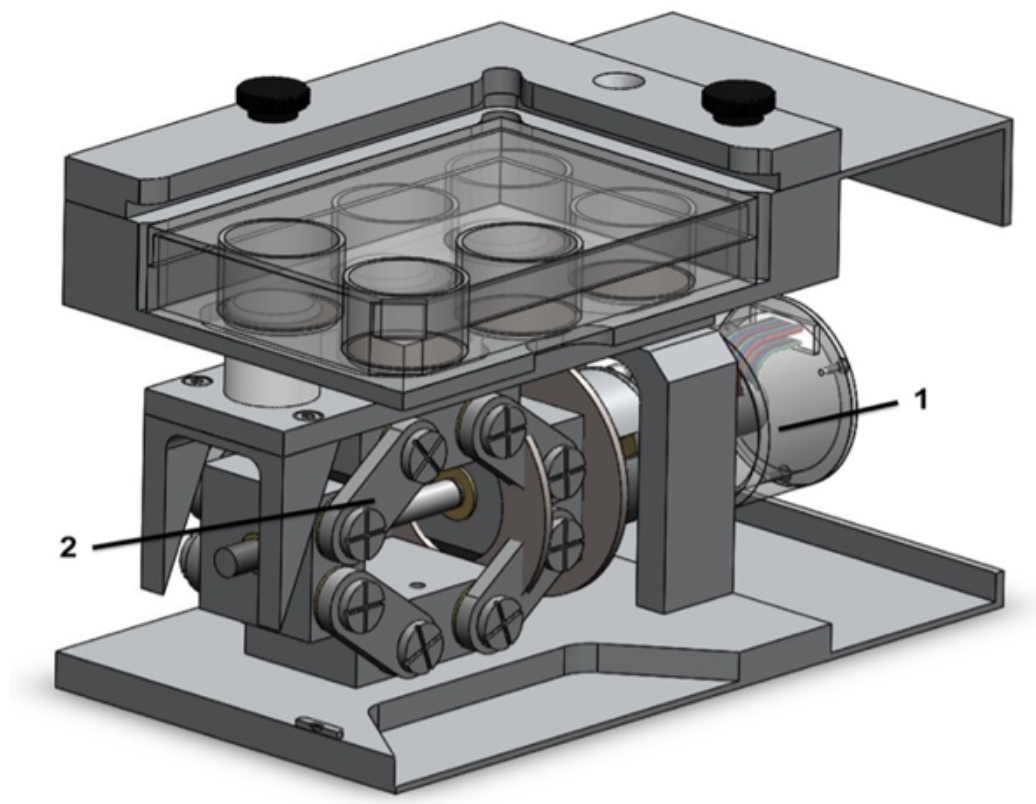

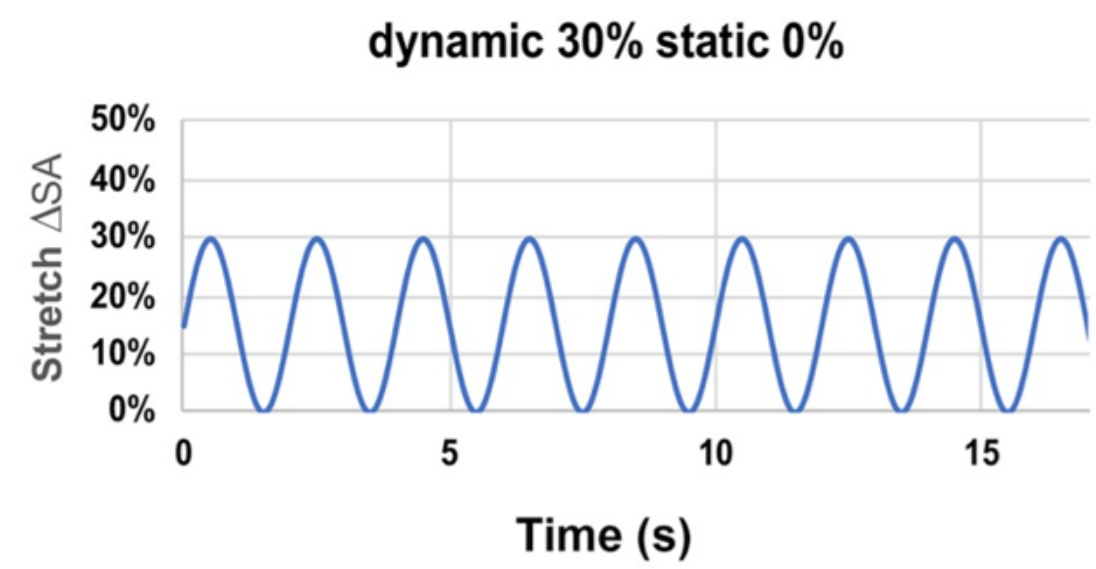

Supplementary Figure 1. – Stretching device. (a) Stretch chamber device used in the experiments with (1) brushless motor driver and (2) vertical cylindrical indenter. (b) Patterns of the tidal stretch ΔSA (estimated change in cell surface area) with the time (s) of L2 AECs performed on the flexible silicone elastomer membrane with a non-variable static stretch pattern of 0%.
