# Supplementary material for: Cyclic stretch increases glucose uptake and pro-inflammatory response in rat alveolar epithelial type II cells
Source: Front Physiol. 2026 Oct 1;17:1909536. doi: 10.3389/fphys.2026.1909536 (PMC13668563; doi:10.3389/fphys.2026.1909536)
Supplement: Supplementary file 2 [file DataSheet2.pdf]

Supplementary Material

Table 2. PCR block table (Pearson).

Block	Time	N	AREG (PCR)	IL6 (PCR)	MCP-1 (PCR)	MIP-2 (PCR)
No LPS	1h	2	N.E.	N.E.	N.E.	N.E.
No LPS	4h	2	N.E.	N.E.	N.E.	N.E.
+LPS	1h	2	N.E.	N.E.	N.E.	N.E.
+LPS	4h	2	N.E.	N.E.	N.E.	N.E.
Stretch vs Control	1h	2	N.E.	N.E.	N.E.	N.E.
Stretch vs Control	4h	2	N.E.	N.E.	N.E.	N.E.
All pooled	1h	4	0.323; p=0.7917	0.024; p=1.0000	-0.168; p=0.7917	0.088; p=0.9583
All pooled	4h	4	0.902; p=0.1250	0.793; p=0.2083	0.816; p=0.2500	0.798; p=0.2500
Stretch only	1h	1	N.E.	N.E.	N.E.	N.E.
Stretch only	4h	1	N.E.	N.E.	N.E.	N.E.
No LPS	1h+4h pooled	4	0.235; p=0.8333	-0.798; p=0.3333	-0.352; p=0.4167	0.862; p=0.1667
+LPS	1h+4h pooled	4	0.724; p=0.3333	0.095; p=0.8750	0.055; p=0.8750	0.311; p=0.6250
All pooled	1h+4h pooled	8	0.368; p=0.4017	-0.032; p=0.9418	-0.040; p=0.9274	0.081; p=0.8426

Table 2. PCR block table (Spearman).

Block	Time	N	AREG (PCR)	IL6 (PCR)	MCP-1 (PCR)	MIP-2 (PCR)

No LPS	1h	2	N.E.	N.E.	N.E.	N.E.
No LPS	4h	2	N.E.	N.E.	N.E.	N.E.
+LPS	1h	2	N.E.	N.E.	N.E.	N.E.
+LPS	4h	2	N.E.	N.E.	N.E.	N.E.
Stretch vs Control	1h	2	N.E.	N.E.	N.E.	N.E.
Stretch vs Control	4h	2	N.E.	N.E.	N.E.	N.E.
All pooled	1h	4	0.600; p=0.4167	-0.400; p=0.7500	-0.400; p=0.7500	0.000; p=1.0000
All pooled	4h	4	0.800; p=0.3333	0.400; p=0.7500	0.200; p=0.9167	0.200; p=0.9167
Stretch only	1h	1	N.E.	N.E.	N.E.	N.E.
Stretch only	4h	1	N.E.	N.E.	N.E.	N.E.
No LPS	1h+4h pooled	4	0.211; p=0.8333	-0.632; p=0.5000	-0.632; p=0.5000	0.632; p=0.5000
+LPS	1h+4h pooled	4	0.400; p=0.7500	0.000; p=1.0000	0.400; p=0.7500	0.000; p=1.0000
All pooled	1h+4h pooled	8	0.263; p=0.5226	-0.240; p=0.5660	-0.096; p=0.8248	0.120; p=0.7797

Table 3. ELISA block table (Pearson)

.

Block	Time	N	IL6 (ELISA)	IL10 (ELISA)	TNF-alpha (ELISA)	MCP-1 (ELISA)	MIP-2 (ELISA)
No LPS	1h	2	N.E.	N.E.	N.E.	N.E.	N.E.
No LPS	4h	2	N.E.	N.E.	N.E.	N.E.	N.E.
+LPS	1h	2	N.E.	N.E.	N.E.	N.E.	N.E.
+LPS	4h	2	N.E.	N.E.	N.E.	N.E.	N.E.
Stretch vs Control	1h	2	N.E.	N.E.	N.E.	N.E.	N.E.

Stretch vs Control	4h	2	N.E.	N.E.	N.E.	N.E.	N.E.
All pooled	1h	4	-0.082; p=0.8333	-0.021; p=0.9583	-0.957; p=0.0833	-0.103; p=0.8333	-0.166; p=0.7083
All pooled	4h	4	0.736; p=0.2917	0.682; p=0.3333	-0.697; p=0.3333	0.621; p=0.5000	0.530; p=0.5417
Stretch only	1h	1	N.E.	N.E.	N.E.	N.E.	N.E.
Stretch only	4h	1	N.E.	N.E.	N.E.	N.E.	N.E.
No LPS	1h+4h pooled	4	-0.484; p=0.6667	-0.247; p=0.8333	-0.881; p=0.0833	-0.786; p=0.2917	0.911; p=0.2083
+LPS	1h+4h pooled	4	-0.384; p=0.6667	0.603; p=0.5000	-0.932; p=0.0417*	-0.599; p=0.5000	-0.715; p=0.3333
All pooled	1h+4h pooled	8	-0.233; p=0.6181	0.161; p=0.6961	-0.856; p=0.0038*	-0.322; p=0.4768	-0.378; p=0.3723

Table 4. ELISA block table (Spearman)

.

Block	Time	N	IL6 (ELISA)	IL10 (ELISA)	TNF-alpha (ELISA)	MCP-1 (ELISA)	MIP-2 (ELISA)
No LPS	1h	2	N.E.	N.E.	N.E.	N.E.	N.E.
No LPS	4h	2	N.E.	N.E.	N.E.	N.E.	N.E.
+LPS	1h	2	N.E.	N.E.	N.E.	N.E.	N.E.
+LPS	4h	2	N.E.	N.E.	N.E.	N.E.	N.E.
Stretch vs Control	1h	2	N.E.	N.E.	N.E.	N.E.	N.E.
Stretch vs Control	4h	2	N.E.	N.E.	N.E.	N.E.	N.E.
All pooled	1h	4	0.000; p=1.0000	-0.400; p=0.7500	-0.800; p=0.3333	0.000; p=1.0000	-0.400; p=0.7500
All pooled	4h	4	0.400; p=0.7500	0.200; p=0.9167	-0.800; p=0.3333	0.200; p=0.9167	0.400; p=0.7500

Stretch only	1h	1	N.E.	N.E.	N.E.	N.E.	N.E.
Stretch only	4h	1	N.E.	N.E.	N.E.	N.E.	N.E.
No LPS	1h+4h pooled	4	0.000; p=1.0000	0.000; p=1.0000	-1.000; p=0.0833	-0.800; p=0.3333	0.800; p=0.3333
+LPS	1h+4h pooled	4	-0.600; p=0.4167	0.400; p=0.7500	-1.000; p=0.0833	-0.600; p=0.4167	-0.600; p=0.4167
All pooled	1h+4h pooled	8	-0.238; p=0.5821	0.048; p=0.9349	-0.905; p=0.0046*	-0.405; p=0.3268	0.000; p=1.0000

Supplementary Figure 2. - Correlation analysis between glucose uptake and inflammatory markers in alveolar epithelial cells exposed to 30% cyclic stretch. Correlation matrices were generated using condition medians from Control, Stretch, LPS, and LPS+Stretch groups at 1 h and 4 h. Analyses were organized into predefined blocks including No LPS, +LPS, Stretch vs Control, Stretch only, and all conditions pooled. Correlations were calculated between glucose uptake and inflammatory markers measured by RT-qPCR (AREG, IL-6, MCP-1, and MIP-2) and ELISA (IL-6, IL-10, TNF-α, MCP-1, and MIP-2). Both Pearson and Spearman correlation coefficients were computed, and exact two-sided permutation tests were used to calculate *p values*. Significant correlations were limited and were observed mainly in pooled and LPS-containing conditions, particularly for TNF-α. No significant PCR-derived inflammatory markers were consistently associated with glucose uptake. These findings suggest that 30% cyclic stretch induces metabolic activation independently of inflammatory signalling, whereas stronger coupling between metabolism and inflammation occurs under LPS-primed condition.
